# Supplementary material for: Evidence for motor imagery in the management of vestibular disorders does not support recent guidelines: A systematic search and review
Source: PLoS One. 2026 Jan 22;21(1):e0337445. doi: 10.1371/journal.pone.0337445 (PMC12826463; doi:10.1371/journal.pone.0337445)
Supplement: S1 Appendix — (DOCX) [file pone.0337445.s001.docx]

**Supplementary Material 1: Search strategy refinement.**

The review protocol was registered a priori in 2023. The initial search strategy for the motor imagery component was adapted from the only previously published review on this topic and reflected the terminology and database indexing available at the time of that review (Schuster, C., Hilfiker, R., Amft, O. et al. Best practice for motor imagery: a systematic literature review on motor imagery training elements in five different disciplines. *BMC Med* 9, 75 (2011). https://doi.org/10.1186/1741-7015-9-75).

The extended duration of the review required updating the searches to ensure currency. Given the low number of studies included and the evolution of terminology and database indexing since the 2011 review, we refined the search syntax to better align with current indexing practices and terminology, while preserving the original conceptual framework of the search and all protocol-defined eligibility criteria.

These refinements did not constitute a change in the research question, scope, or review methods. Rather, they represent an operational optimization of how predefined concepts were translated into database-specific search syntax. We deliberately chose not to amend the registered protocol, as the review methods remained unchanged, and the review had progressed beyond study selection and data extraction. Retrospective protocol modification to reflect search syntax refinements alone could introduce ambiguity regarding selective methodological adaptation.

The refined search strategy retrieved fewer records than the initial strategy, reflecting improved specificity rather than reduced coverage. All records retrieved across search iterations were deduplicated and screened using identical eligibility criteria and procedures. The refined strategy did not identify additional eligible studies beyond those already included, suggesting that the initial strategy had already achieved adequate coverage of the available evidence base and that the refinements served to confirm, rather than alter, the review findings.

To ensure transparency and reproducibility, the full search strategies for each database and search iteration are reported below.

01/06/2025

PubMed : n=48

("Vestibular System"[Mesh] OR "Vestibular Diseases"[Mesh] OR semicircul* OR vestibul* OR labyrinth* OR vertigo OR otolith* OR "hair cell*" OR ampulla OR "oval window*" OR sacul* OR utricul*) AND ("Imagery, Psychotherapy"[Mesh] OR "motor imager*" OR "mental imager*" OR "mental practic*" OR "motor practic*" OR "mental simulat*" OR "motor simulat*")

Scopus: n=2288

ALL ( ( semicircul* OR vestibul* OR labyrinth* OR vertigo OR otolith* OR "hair cell*" OR ampulla OR "oval window*"OR sacul* OR utricul* ) AND ( "motor imager*" OR "mental imager*" OR "mental practic*" OR "motor practic*" OR "mental simulat*" OR "motor simulat*" ) )

CINAHL: n=110

TX ((semicircul* OR vestibul* OR labyrinth* OR vertigo OR otolith* OR "hair cell*" OR ampulla OR "oval window*" OR sacul* OR utricul*) AND ("motor imager*" OR "mental imager*" OR "mental practic*" OR "motor practic*" OR "mental simulat*" OR "motor simulat*"))

Web Of science: n=83

(ALL=(semicircul* OR vestibul* OR labyrinth* OR vertigo OR otolith* OR "hair cell*" OR ampulla OR "oval window*"OR sacul* OR utricul*)) AND ALL=("motor imager*" OR "mental imager*" OR "mental practic*" OR "motor practic*" OR "mental simulat*" OR "motor simulat*")

Cochrane Library: n=14

#1: MeSH descriptor: [Vestibular Diseases] explode all trees

#2: MeSH descriptor: [Vestibular Systems] explode all trees

#3: MeSH descriptor: [Imagery, Psychotherapy] explode all trees

#4: (semicircular OR semi-circular OR vestibular OR vestibule OR labyrinth OR labyrinthine OR vertigo OR otolith OR otolithic OR "hair cell" OR "hair cells" OR ampulla OR ampullary OR "oval window" OR "oval windows" OR saccule OR sacculi OR utricle OR utricular)

#5: "motor imagery" OR "motor imageries" OR "mental imagery" OR "mental imageries" OR "mental practice" OR "mental practices" OR "motor practice" OR "motor practices" OR "mental simulation" OR "mental simulations" OR "motor simulation" OR "motor simulations"

#6: (#1 OR #2 OR #4) AND (#3 OR #5)
