# Supplementary material for: Evidence for motor imagery in the management of vestibular disorders does not support recent guidelines: A systematic search and review
Source: PLoS One. 2026 Jan 22;21(1):e0337445. doi: 10.1371/journal.pone.0337445 (PMC12826463; doi:10.1371/journal.pone.0337445)
Supplement: S2 Appendix — (DOCX) [file pone.0337445.s002.docx]

Table 1: Additional material explaining the choice of analysis criteria in the critical grid.

|  | **Why was this criteria selected?** |
| --- | --- |
| **External validity** | **Applicability of the results to other populations.** |
| Representative sample | **Applicability to the clinical population usually seen by the clinical readers.** |
| Material cost | **Important elements to assess ecological validity. With these different criteria, clinicians can directly identify the applicability of the interventions in their own setting.** |
| Assessment duration |  |
| Intervention duration  feasibility element (tools, recruitment)  Mental imagery instruction |  |
| **Statistical validity** | **Necessary for a critical reading of the article.** |
| Statistical power |  |
| Fishing |  |
| **Internal validity** |  |
| Randomization |  |
| Blinding |  |
| Control |  |
| Evidence of measurement properties |  |
| **Recommendations for motor imagery practice** | **We based our analysis on the article by Schuster et al. [31], which propose “best practice for motor imagery: a systematic literature review on motor imagery training elements in five different disciplines”** |
| Motor imagery capacity evaluation | **Poor motor imaging capabilities may limit the motor imaging program. It is important to be aware of the capabilities of the individuals included in order to ensure that the technique is being administered to subjects who can benefit from it.** |
| Perspective information  (internal or external) | **The perspective and modality of the practice will influence the impact of motor imagery. External and internal perspective programs do not engage the same regions of the central nervous system. This point is key to understanding the program delivered and reproducing it. It is also possible (and desirable) to evolve from an external perspective in visual modality to an internal perspective in kinesthetic modality.** |
| Perspective evolution |  |
| Modality information  (visual or kinesthesic) |  |
| Modality evolution |  |
| Detailed MI instructions | **The detailed information provides an understanding of the program, its regularity, intensity, duration, and the tools used. It is important to have these elements in order to replicate the program (for research or therapy purposes).** |
| Familiarisation | **Familiarization is recommended, as motor imagery is still a cognitive therapy that is not always common practice. This familiarization allows for a standardized approach with patients/subjects.** |
| Closed eyes | **Opening the eyes can provide visual cues that help to perfect the motor imagery produced. It is important to clarify this point for reproducibility.** |
| **Recommendations for vestibular disorders practice** | **We based our analysis on the reviews by Hall et al. [6], the recommendations of the Barany Society, and the French recommendations for vestibular rehabilitation [https://www.sforl.org/wp-content/uploads/2023/08/Reco-Place-de-la-reeducation-dans-la-prise-en-charge-des-vertiges-dorigine-vestibulaire-16-08-2023.pdf ].** |
| Rotary chair | **Rotary chair testing provides a highly reliable measure of horizontal semicircular canal function across multiple frequencies. Its central role is comparable to "capacity evaluation" in MI: it informs whether the vestibular system is capable of participating in adaptation.** |
| Optokinetic stimulation | **Optokinetic stimuli challenge visual motion processing and visual dependence—similar to “modality information” in MI** |
| Vestibular stimulation exercises: | **These exercises target vestibular adaptation mechanisms, similar to “modality evolution” or “perspective evolution” in MI. They must be clearly detailed for replication.** |
| Vestibular habituation exercises: | **Habituation reduces symptom provocation through repeated exposure—this parallels the “familiarisation” concept in MI.** |
| Balance retraining exercises: | **Balance tasks train sensory reweighting, analogous to ensuring correct MI** perspective **or** modality **for task specificity.** |
| Eyes movement exercises | **Oculomotor control influences VOR engagement and visual tracking—similar to “detailed instructions” in MI.** |
| Head and eye coordination | **This is equivalent to combining** perspective + modality **in MI: the task integrates multisensory and motor components.** |
