# Supplementary material for: Evidence for motor imagery in the management of vestibular disorders does not support recent guidelines: A systematic search and review
Source: PLoS One. 2026 Jan 22;21(1):e0337445. doi: 10.1371/journal.pone.0337445 (PMC12826463; doi:10.1371/journal.pone.0337445)
Supplement: Attachment — Submitted filename: Rebuttal letter_V4.docx [file pone.0337445.s003.docx]

Dear college,

thank you for your attention and comments of our study.

1. Our submission PONE-D-24-54538 was rejected for the following reason:

*« PLOS ONE considers systematic reviews and meta-analyses for publication; however, these submissions must address a clear and defined research question; conduct a systematic and comprehensive literature review; and use clearly reported, reproducible, and systematic methods to identify, select, and critically appraise relevant research. Our author guidelines for systematic reviews are at https://journals.plos.org/plosone/s/submission-guidelines#loc-systematic-reviews-and-meta-analyses.*

*In the case of your submission, we are concerned that the literature review was completed in August 2023. Systematic and scoping reviews should provide an up-to-date overview of the research topic covered. Since the literature search was completed over a year ago, we are concerned that this study does not meet this requirement. »*

In order to enable a re-evaluation of this work, which we consider important for clinical practice and scientific reflection, we have therefore provided an update on June 1, 2025, less than a month ago to date.

2. Regarding the update submitted under number PONE-D-25-34740, the comments are as follows :

*« 1. It has come to our attention that the manuscript seems to be a resubmission of the manuscript “PONE-D-24-54538”, which was previously considered by the journal. It is a requirement by the journal that any manuscript resubmitted after a previous rejection is accompanied by a rebuttal letter outlining the response to the original reviewers' comments, so that the editors can establish whether the original concerns have been addressed. Can you therefore please provide us with a point-by-point response to the comments provided by the original reviewers, outlining the revisions carried out since the original submission. Please upload this as "other" in the file inventory.

2. It appears that you did not upload a Track Changes version of your manuscript file, which highlights any changes you made to your previous submission (PONE-D-24-54538). Authors must submit Track Changes MS files as required by the journal on new submissions of previous PLOS ONE MSs. At this time, please upload a Track Changes version of your manuscript to your File Inventory as an ‘Other’ file. »*

Thank you for your additional comments. We have therefore written a rebutal letter, in addition to the explanations in the letter to the editor. A supplementary document with Track Changes version of our manuscript has been added to the documents.

3. Regarding the update submitted under number PONE-D-25-34740, the comments are as follows :

*«  PLOS ONE considers systematic reviews and meta-analyses for publication; however, these submissions must address a clear and defined research question; conduct a systematic and comprehensive literature review; and use clearly reported, reproducible, and systematic methods to identify, select, and critically appraise relevant research. Our author guidelines for systematic reviews are at* [*https://journals.plos.org/plosone/s/submission-guidelines#loc-systematic-reviews-and-meta-analyses*](https://urldefense.com/v3/__https:/journals.plos.org/plosone/s/submission-guidelines*loc-systematic-reviews-and-meta-analyses__;Iw!!E0Ahi1ckbONv5w!9EjGfrUw-yVUr4TX7qgoW904kCAYyiECUIx-Ygbk-qq19kcH8tS6flFf_8VhXQMNb3ZUP-v7tM_Uy3_-onaCLQ-qbQ$)*.
 
In this case, we note that there is no assessment of the certainty of the body of evidence for each outcome. This is distinct from assessments of the quality or risk of bias of the included studies, and is essential to inform further healthcare decisions based on the results of the review. We do not feel that valid conclusions can be drawn from your review without this assessment  (for more information, please see Schünemann HJ, Higgins JPT, Vist GE, Glasziou P, Akl EA, Skoetz N, Guyatt GH. Chapter 14: Completing ‘Summary of findings’ tables and grading the certainty of the evidence [last updated August 2023]. In: Higgins JPT, Thomas J, Chandler J, Cumpston M, Li T, Page MJ, Welch VA (editors). Cochrane Handbook for Systematic Reviews of Interventions version 6.5. Cochrane, 2024. Available from* [*www.training.cochrane.org/handbook*](https://urldefense.com/v3/__http:/www.training.cochrane.org/handbook__;!!E0Ahi1ckbONv5w!9EjGfrUw-yVUr4TX7qgoW904kCAYyiECUIx-Ygbk-qq19kcH8tS6flFf_8VhXQMNb3ZUP-v7tM_Uy3_-onaykblGqQ$) *).*

*In addition, we note that language restrictions are imposed on the literature searches without adequate justification. There is increasing recognition that exclusion of studies published in other languages can increase the risk of bias in systematic reviews. We feel that any restrictions should be justified based on scientific reasons rather than resource constraints. As a result, we have concerns about the comprehensiveness of the review and the validity of the conclusions »*

Regarding your first comment : Our manuscript follows the “systematic search and review” typology as defined by Grant and Booth (2009). This approach is particularly suited to exploring emerging or under-researched topics—such as motor imagery in vestibular rehabilitation—as it combines a structured and comprehensive search process with a descriptive and interpretive synthesis of the evidence. Within this framework, the use of a formal risk of bias assessment and a certainty-of-evidence rating (such as GRADE) is not a required component, especially when the goal is not to quantify effects but to map and critically reflect on the available literature. In our review, we sought to provide a structured synthesis of existing studies on motor imagery in the context of vestibular rehabilitation. To do so, we developed a tailored critical appraisal grid that allowed us to examine each study based on methodological elements, feasibility, and alignment with research best practices in both motor imagery and vestibular rehabilitation. This appraisal provided important insights into the strengths and limitations of current research and supported the identification of meaningful directions for future work. For instance, our appraisal highlighted that none of the included studies assessed motor imagery ability, despite the fact that this element is widely recognized as essential in motor imagery research. This omission limits the interpretability and reproducibility of the interventions studied. By identifying such gaps, the appraisal contributes to the development of more robust and clinically useful research in this area. Lastly, we would like to highlight that a member of our team has previously applied and published this methodology in a Q1-ranked journal (Impact Factor 2.9), reinforcing its relevance and acceptance in high-quality scientific outlets. The article has received notable attention, with over 8,000 accesses, an Altmetric Attention Score of 27, and was featured as "Paper of the Week" by the International Association for the Study of Pain ([https://doi.org/10.1186/s40945-021-00109-y](https://urldefense.com/v3/__https:/doi.org/10.1186/s40945-021-00109-y__;!!E0Ahi1ckbONv5w!9zQ6aWfVUMeSpoGgv1RmedUMOiv7_8StEdQfYXd_7G-sRcef-gJfElw5tj1dpNgD6L42b5zYQmrXbhA85GaK7Hf4fds4ocPq4JuTxeo$)). We hope this explanation helps clarify our approach and we remain available should you need any further detail.

Regarding your second comment, we have updated the process. The paper previously excluded because it was not in English has been excluded for an other reason (not concerning motor imagery intervention). As a result, there has been no change in the results of our search.

Kind regards

Thomas Rulleau for the co-authors
