# Supplementary material for: Evidence for motor imagery in the management of vestibular disorders does not support recent guidelines: A systematic search and review
Source: PLoS One. 2026 Jan 22;21(1):e0337445. doi: 10.1371/journal.pone.0337445 (PMC12826463; doi:10.1371/journal.pone.0337445)
Supplement: Attachment — Submitted filename: rebuttal_letter_Cachalot_V5.docx [file pone.0337445.s004.docx]

|  | **Academic editor comments** | *Response* | **Modification in manuscript** |
| --- | --- | --- | --- |
|  | **Please see the reviewer's comments, respond and revise the manuscript as needed.** | *Dear colleague, we hope we have answered all the experts' questions. Thank you for your interest in our work.* |  |
| **Reviewer 1** | | |  |
| N° | **Comments** | *Response* | **Modification in manuscript** |
| 1 | The manuscript follows a systematic search and review approach, which is appropriate for emerging or under-researched topics but here few comments to consider | *We appreciate your interest and hope to clarify your request.* |  |
| 2 | Explicitly compare “systematic search and review” vs. systematic review, and explain why GRADE or equivalent was not applied | *We modified the manuscript accordingly.* | **Lines 100 to 105**  “Unlike traditional systematic reviews, which aim to evaluate intervention effectiveness and therefore apply tools such as GRADE to rate the certainty of effect estimates, a systematic search and review focuses on describing interventions, examining their clinical applicability, and identifying conceptual or methodological gaps ([https://onlinelibrary.wiley.com/doi/full/10.1111/j.1471-1842.2009.00848.x](https://urldefense.com/v3/__https:/onlinelibrary.wiley.com/doi/full/10.1111/j.1471-1842.2009.00848.x__;!!E0Ahi1ckbONv5w!4yDCjfUO7YEk6uV6IU21XX315vg6T16yr1JIc3KN2ojWauyUluU0nL0hauQ9k51ExTIe1xMnZ1yAzBwgqp26v3dgjUtsbd7zs2Biv7E$) ; [https://pubmed.ncbi.nlm.nih.gov/40914296/](https://urldefense.com/v3/__https:/pubmed.ncbi.nlm.nih.gov/40914296/__;!!E0Ahi1ckbONv5w!4yDCjfUO7YEk6uV6IU21XX315vg6T16yr1JIc3KN2ojWauyUluU0nL0hauQ9k51ExTIe1xMnZ1yAzBwgqp26v3dgjUtsbd7zGSPhLfI$)). In this context, GRADE or equivalent frameworks, which are designed to assess confidence in pooled or comparative effect estimates, are not appropriate ([https://www.sciencedirect.com/science/article/pii/S2213398423002713](https://urldefense.com/v3/__https:/www.sciencedirect.com/science/article/pii/S2213398423002713__;!!E0Ahi1ckbONv5w!4yDCjfUO7YEk6uV6IU21XX315vg6T16yr1JIc3KN2ojWauyUluU0nL0hauQ9k51ExTIe1xMnZ1yAzBwgqp26v3dgjUtsbd7zqkNyvhk$)).  .” |
| 3 | The appraisal tool is not benchmarked against established checklists (e.g., ROBINS-I, Cochrane Risk of Bias). | *We modified the manuscript accordingly.*  *A detailed explanation of this methodological choice is available below:*  *Because our review is a systematic search and review rather than a systematic review of intervention effectiveness, our purpose was not to estimate causal effects or formally compare trial outcomes. Instead, our objectives were to (1) describe the vestibular interventions used in clinical trials; (2) assess their clinical applicability and methodological limitations using a critical grid grounded in best practice in motor imagery and vestibular rehabilitation; and (3) summarize trial outcomes only to illustrate that current recommendations may overinterpret the evidence base.*  *For this type of big-picture review, established risk-of-bias tools such as ROBINS-I or RoB-2, which focus on internal validity for causal inference, are not appropriate or required. They do not evaluate intervention content, clinical applicability, or adherence to best practice principles. Our tailored appraisal grid is therefore aligned with the methodological intent of systematic search and review approaches and with our research question.* | **Lines 145 to 151**  “One objective of a systematic search and review is to provide practice-oriented insights rather than to appraise internal validity for causal inference ([https://onlinelibrary.wiley.com/doi/full/10.1111/j.1471-1842.2009.00848.x](https://urldefense.com/v3/__https:/onlinelibrary.wiley.com/doi/full/10.1111/j.1471-1842.2009.00848.x__;!!E0Ahi1ckbONv5w!4yDCjfUO7YEk6uV6IU21XX315vg6T16yr1JIc3KN2ojWauyUluU0nL0hauQ9k51ExTIe1xMnZ1yAzBwgqp26v3dgjUtsbd7zs2Biv7E$)). Accordingly, we developed a tailored critical appraisal tool to evaluate the external validity, feasibility, and alignment of intervention components with current best practice in motor imagery and vestibular rehabilitation. This grid was designed to capture aspects of intervention content and clinical applicability that are not addressed by traditional risk-of-bias tools. Key elements of contemporary motor imagery and vestibular practice were therefore integrated to support a clinically meaningful interpretation of the included trials.” |
| 4 | No quantitative synthesis or effect-size estimation; conclusions as underpowered. | *We did not conduct a quantitative synthesis because it was not aligned with the objectives of this systematic search and review. Although quantitative synthesis without meta-analysis is feasible, for example using the SWiM framework, such approaches remain appropriate only when the aim is to summarise effect estimates across studies. In our case, the primary objective was to describe intervention content, assess clinical applicability, and examine alignment with best practice in motor imagery and vestibular rehabilitation, rather than to quantify treatment effects. With only two included studies and substantial differences in intervention content, participants, and outcome constructs, applying a structured quantitative synthesis would have added little interpretive value and risked overstating the strength of the evidence. The absence of quantitative synthesis therefore does not render our conclusions “underpowered”; instead, it reflects the limited and heterogeneous evidence base and the practice-oriented focus of this review.* |  |
| *Reviewer 2* | | |  |
|  | **Comments** | *Response* | **Modification in manuscript** |
| 1 | **The manuscript is relevant, timely, and methodologically transparent. However, before publication, the authors should:** | *We would like to thank reviewer 2 for their comments on the value of our work.* |  |
| 2 | **Provide a more explicit assessment of certainty in evidence (even if narrative).** | *Table 2 illustrated the assesment. We had pointed out that a lot of data was missing. We have added a more explicit assessment, as requested.* | **Lines 192 -195 :**  **« The data collected show low to no external validity, unrepresentative or poorly adapted assessment, lack of statistical validity, low internal validity, lack of use of motor imagery intervention recommendations, and lack of use of recommendations for practice in vestibular disorders.”** |
| 3 | **Clarify and justify the language restrictions.** | *We restricted our search to studies published in English and French. These are the working languages of the review team, allowing accurate screening, data extraction, and interpretation without requiring external translation resources. Although the Cochrane Handbook recommends including studies in all languages to minimise selection bias, applying this principle requires access to reliable translation capacity. Using automated translation technologies may appear to overcome this barrier, but it carries risks of mistranslation, particularly for clinically nuanced terminology, intervention components, and methodological details, which could compromise the accuracy of data extraction and appraisal. In vestibular rehabilitation and motor imagery, where the vast majority of trials are published in English, this restriction is unlikely to have materially affected study retrieval or the conclusions.* |  |
| 4 | **Reframe conclusions with stronger caution regarding clinical applicability.** | *We appreciate your comment and have amended the conclusion accordingly.* | **Lines 283-287 :**  **“This systematic search and review identified two studies evaluating the efficacy of motor imagery interventions for the rehabilitation of vestibular disorders. While studies suggest that motor imagery rehabilitation reduces the vestibulo-ocular reflex, there are some methodological concerns included the small sample size, absence of imagery quality assessment, and poor generalizability. However, although motor imagery appears to be affected by vestibular pathology, the paucity and lack of quality of the available evidence does not allow us to recommend its use in routine practice at this time.”** |
| 5 | **Improve clarity in reporting of intervention details and future research needs.** | *We appreciate your comment and have amended the conclusion accordingly.* | **Lines 263 to 271 :**  **« Specifically, future research should include: 1. assessment of subjects' motor imaging quality (vestibular damage may affect imaging quality); 2. a battery of vestibular tests (Video Head Impulse Test assessment, quality of life as measured by the Dizziness Handicap Inventory); 3. a clear, reproducible, and realistic rehabilitation program for use in routine practice, in accordance with recommendations on motor imaging [29] and vestibular rehabilitation [6]. For example, it should include motor imagery exercises related to the practice of vestibular rehabilitation therapists, with imagery reproduction of these exercises, of a specific duration, intensity, and regularity. The number of subjects required should be determined based on a primary endpoint predefined before the study. The study must be multicenter in order to avoid a “center effect. Given the heterogeneity of vestibular pathologies, future studies should focus on a specific vestibular pathology (e.g., neuritis, Meniere's syndrome, etc.) to test these hypotheses. Finally, studies on healthy subjects receiving vestibular stimulation could be conducted.”.** |
| 6 | **If these revisions are addressed, the manuscript would represent a valuable contribution to the literature on vestibular rehabilitation and motor imagery** | *We would like to thank reviewer 2 for their comments on the contribution of our work on vestibular rehab and motor imagery* |  |
