# Supplementary material for: Evidence for motor imagery in the management of vestibular disorders does not support recent guidelines: A systematic search and review
Source: PLoS One. 2026 Jan 22;21(1):e0337445. doi: 10.1371/journal.pone.0337445 (PMC12826463; doi:10.1371/journal.pone.0337445)
Supplement: Attachment — Submitted filename: rebuttal_letter_Cachalot_V6.docx [file pone.0337445.s005.docx]

|  | **Academic editor comments** | *Response* | **Modification in manuscript** |
| --- | --- | --- | --- |
|  | The manuscript will be strengthened if the authors consider the following points. | *We thank the academic editor for this comment and for his interest in our work.* |  |
|  | 1. Authors provide a link to the search criteria used (supplemental material to their PROSPERO submission), which also includes results, in terms of number of articles identified from a search conducted in August 2023. It appears for this manuscript, authors redid the search in June 2025. However, the number of articles identified using the same search criteria, in some cases, are quite a bit less than that identified in 2023 which does not make sense to me (for example, in 2023, PubMed identified 201 articles, but according to Figure 1, the current search only identified 48 from PubMed; similarly in 2023, CINAHL identified 33 articles, while the current search identified 9 and Cochrane library identified 70 in 2023 and only 5 in 2025). Authors should clarify reasons why the current search resulted in fewer articles from the same sources using the same criteria. If criteria were modified, that needs to be clarified. | *Thank you for this comment. Here is the justification provided in the newly developed supplementary material.*  *The review protocol was registered a priori in 2023. The initial search strategy for the motor imagery component was adapted from the only previously published review on this topic and reflected the terminology and database indexing available at the time of that review (Schuster, C., Hilfiker, R., Amft, O. et al. Best practice for motor imagery: a systematic literature review on motor imagery training elements in five different disciplines. BMC Med 9, 75 (2011). https://doi.org/10.1186/1741-7015-9-75).*  *The extended duration of the review required updating the searches to ensure currency. Given the low number of studies included and the evolution of terminology and database indexing since the 2011 review, we refined the search syntax to better align with current indexing practices and terminology, while preserving the original conceptual framework of the search and all protocol-defined eligibility criteria.*  *These refinements did not constitute a change in the research question, scope, or review methods. Rather, they represent an operational optimization of how predefined concepts were translated into database-specific search syntax. We deliberately chose not to amend the registered protocol, as the review methods remained unchanged, and the review had progressed beyond study selection and data extraction. Retrospective protocol modification to reflect search syntax refinements alone could introduce ambiguity regarding selective methodological adaptation.*  *The refined search strategy retrieved fewer records than the initial strategy, reflecting improved specificity rather than reduced coverage. All records retrieved across search iterations were deduplicated and screened using identical eligibility criteria and procedures. The refined strategy did not identify additional eligible studies beyond those already included, suggesting that the initial strategy had already achieved adequate coverage of the available evidence base and that the refinements served to confirm, rather than alter, the review findings.*  *To ensure transparency and reproducibility, the full search strategies for each database and search iteration are reported in the supplementary material.*  *Questions on this point led us to review our update as well. We apologize for the error that occurred during the last submission, where one of the databases was not queried correctly. We have made the correction and verified everything. There is therefore a discrepancy of 60 references with this correction.* | **Line 144: We added: “See Supplementary Material 1 for the full search strategy and justification of search strategy refinements following protocol registration”.**  **Line 45 : “2404”**  **Line 165 : 2543**  **Line 166 :“2404”** |
|  | 2. Given that authors created their own set of criteria for the clinical evaluation of the interventions, authors should provide in supplemental material what each criterion represents and what information they were looking for (give examples). | *We thank you for your comments. We have added supplementary material on these points, which will give consistency to our work.* | **Line 191: we added : “See the supplementary material for explanations of the critical grid.”** |
|  | 1. line 75: there is a missing "." at the end of the sentence (between "functions" and "Yardley"). | *thanks* | **Line 75 : We added a dot between “functions” and “Yardley”** |
|  | 2. line 82: change "affect" to "affects" | *thanks* | **Line 82: we changed "affect" to "affects"** |
|  | 3. line 113: there is an error with the reference (Error! Reference source not found) | *We have no “error” on our version. Il could be a compatibility problem.*  *Ref 23 : 23. Schiavenato M, Chu F. PICO: What it is and what it is not. Nurse Educ Pract. 2021;56: 103194. doi:10.1016/j.nepr.2021.103194* |  |
|  | 4. line 164: "1 more articles" should be "1 more article" | *thanks* | **Line 164, we modified: "1 more articles" to "1 more article"** |
|  | 5. line 183: authors mention 9 females in the Nigmatullina study, but this does not match what is presented in Table 1 - looking up the reference, it appears as though there were 2 experiments with different number of females in each experiment - it may be that authors only utilized information from 1 experiment, but that should be clarified. | *Thanks* | **Line 183, we added “nine (experiment 1) + seven (experiment 2)”, in order to clarify.** |
| **Reviewer 3** | | |  |
| N° | **Comments** | *Response* | **Modification in manuscript** |
| 1 | The authors present a systematic search and review of motor imagery interventions used as part of vestibular rehabilitation, somewhat in response to an article that encouraged the use of motor imagery in rehabilitation. Authors felt this recommendation was potentially premature and wanted to review what information existed about the use of motor imagery. With only 2 studies identified that met their inclusion criteria, authors evaluated the information contained in the publications regarding the intervention and quality of evidence using a set of criteria they identified as being relevant and important. The overall conclusion was that evidence was not yet present to support active use of motor imagery in rehabilitation, but encouraged more research to be done to evaluate its impact on clinical measures. The manuscript will be strengthened if the authors consider the following points. | *We thank the reviewer 3 for this comment and for his interest in our work.* |  |
| 2 | 1. Authors provide a link to the search criteria used (supplemental material to their PROSPERO submission), which also includes results, in terms of number of articles identified from a search conducted in August 2023. It appears for this manuscript, authors redid the search in June 2025. However, the number of articles identified using the same search criteria, in some cases, are quite a bit less than that identified in 2023 which does not make sense to me (for example, in 2023, PubMed identified 201 articles, but according to Figure 1, the current search only identified 48 from PubMed; similarly in 2023, CINAHL identified 33 articles, while the current search identified 9 and Cochrane library identified 70 in 2023 and only 5 in 2025). Authors should clarify reasons why the current search resulted in fewer articles from the same sources using the same criteria. If criteria were modified, that needs to be clarified. | *Thank you for this comment. Here is the justification provided in the newly developed supplementary material.*  *The review protocol was registered a priori in 2023. The initial search strategy for the motor imagery component was adapted from the only previously published review on this topic and reflected the terminology and database indexing available at the time of that review (Schuster, C., Hilfiker, R., Amft, O. et al. Best practice for motor imagery: a systematic literature review on motor imagery training elements in five different disciplines. BMC Med 9, 75 (2011). https://doi.org/10.1186/1741-7015-9-75).*  *The extended duration of the review required updating the searches to ensure currency. Given the low number of studies included and the evolution of terminology and database indexing since the 2011 review, we refined the search syntax to better align with current indexing practices and terminology, while preserving the original conceptual framework of the search and all protocol-defined eligibility criteria.*  *These refinements did not constitute a change in the research question, scope, or review methods. Rather, they represent an operational optimization of how predefined concepts were translated into database-specific search syntax. We deliberately chose not to amend the registered protocol, as the review methods remained unchanged, and the review had progressed beyond study selection and data extraction. Retrospective protocol modification to reflect search syntax refinements alone could introduce ambiguity regarding selective methodological adaptation.*  *The refined search strategy retrieved fewer records than the initial strategy, reflecting improved specificity rather than reduced coverage. All records retrieved across search iterations were deduplicated and screened using identical eligibility criteria and procedures. The refined strategy did not identify additional eligible studies beyond those already included, suggesting that the initial strategy had already achieved adequate coverage of the available evidence base and that the refinements served to confirm, rather than alter, the review findings.*  *To ensure transparency and reproducibility, the full search strategies for each database and search iteration are reported in the supplementary material.*  *Questions on this point led us to review our update as well. We apologize for the error that occurred during the last submission, where one of the databases was not queried correctly. We have made the correction and verified everything. There is therefore a discrepancy of 60 references with this correction.* | **Line 144: We added:**  **“See Supplementary Material 1 for the full search strategy and justification of search strategy refinements following protocol registration”.**  **Line 45 : “2404”**  **Line 165 : 2543**  **Line 166 :“2404”** |
| 3 | 2. Given that authors created their own set of criteria for the clinical evaluation of the interventions, authors should provide in supplemental material what each criterion represents and what information they were looking for (give examples). | *We thank you for your comments. We have added supplementary material on these points, which will give consistency to our work.* |  |
| 4 | 1. line 75: there is a missing "." at the end of the sentence (between "functions" and "Yardley"). | *thanks* |  |
| 5 | 2. line 82: change "affect" to "affects" | *thanks* |  |
| 6 | 3. line 113: there is an error with the reference (Error! Reference source not found) | *We have no “error” on our version. Il could be a compatibility problem.*  *Ref 23 : 23. Schiavenato M, Chu F. PICO: What it is and what it is not. Nurse Educ Pract. 2021;56: 103194. doi:10.1016/j.nepr.2021.103194* |  |
| 7 | 4. line 164: "1 more articles" should be "1 more article" | *thanks* |  |
| 8 | 5. line 183: authors mention 9 females in the Nigmatullina study, but this does not match what is presented in Table 1 - looking up the reference, it appears as though there were 2 experiments with different number of females in each experiment - it may be that authors only utilized information from 1 experiment, but that should be clarified. | *Thanks* |  |
